# Interpreting and assessing confidence in network meta-analysis results: an introduction for clinicians

**DOI:** 10.1007/s00540-022-03072-5

**Published:** 2022-06-01

**Authors:** Alan Yang, Petros Pechlivanoglou, Kazuyoshi Aoyama

**Affiliations:** 1 Program in Child Health Evaluative Sciences, The Hospital for Sick Children Research Institute, Toronto, Canada; 2 Department of Anesthesia and Pain Medicine, The Hospital for Sick Children, 555 University Ave, #2211, Toronto, ON M5G 1X8 Canada

**Keywords:** Network meta-analysis, Indirect treatment comparisons, Multiple treatment comparisons, Credible intervals, Confidence intervals

## Abstract

**Purpose:**

We aimed to provide clinicians with introductory guidance for interpreting and assessing confidence in on Network meta-analysis (NMA) results.

**Methods:**

We reviewed current literature on NMA and summarized key points.

**Results:**

Network meta-analysis (NMA) is a statistical method for comparing the efficacy of three or more interventions simultaneously in a single analysis by synthesizing both direct and indirect evidence across a network of randomized clinical trials. It has become increasingly popular in healthcare, since direct evidence (head-to-head randomized clinical trials) are not always available. NMA methods are categorized as either Bayesian or frequentist, and while the two mostly provide similar results, the two approaches are theoretically different and require different interpretations of the results.

**Conclusions:**

We recommend a careful approach to interpreting NMA results and the validity of an NMA depends on its underlying statistical assumptions and the quality of the evidence used in the NMA.

## Introduction

The highest level of evidence for the comparative effectiveness of different clinical interventions generally comes from systematic reviews of randomized controlled trials (RCTs) [1–3]. The most conventional and widely used method for synthesizing the results of different RCTs is pairwise meta-analysis [4, 5]. While this statistical approach is useful, it is limited as it can only compare two interventions at a time, and only head-to-head RCTs that involve the comparison of interest [6].

Network meta-analysis (NMA) is a statistical method that extends the principles of pairwise meta-analysis to the evaluation of multiple interventions in a single process, which is achieved by combining both direct and indirect evidence [4, 5, 7, 8]. Direct evidence represents evidence obtained from head-to-head RCTs [4]. For example, in an RCT comparing interventions A and B, the estimate of relative effectiveness of A versus B counts as direct evidence. Indirect evidence represents evidence obtained from one or more common comparators; for example, in the absence of RCTs that evaluate interventions A and B directly, interventions A and B can be indirectly compared if both have been compared to a common intervention C in existing trials [4]. The combination of direct and indirect evidence is at the core of a network meta-analysis [5, 7, 8].

Network meta-analysis is a statistical method for synthesizing direct and indirect evidence from a network of clinical trials to concurrently compare multiple clinical interventions in a single process [4, 5, 7–9]. Synonymous names of NMA include multiple treatment meta-analysis, indirect treatment comparisons, and mixed treatment comparisons [1, 10]. NMA has become attractive among clinicians and health-care researchers in recent years because of its ability to evaluate the comparative clinical effectiveness of different clinical interventions based on clinical evidence through a robust quantitative framework [3, 8, 11]. However, due to its complex structure and methodological requirements, a careful approach is required when interpreting NMA results, to avoid drawing biased or incorrect conclusions [3, 12]. This article aims to provide clinicians with introductory guidance for interpreting and assessing confidence in NMA results.

## Interpretation of NMA results

NMA has matured over the recent years and NMA models are available for different types of individual-level and trial-level data and summary effect measures (e.g., odds ratio, risk difference) and are being implemented in both frequentist and Bayesian frameworks [2, 13, 14]. Typically, interventions are displayed in the form of a network, called a network diagram. Statistical approaches to NMA are broadly classified as frequentist and Bayesian frameworks [1, 2, 15]. The Bayesian framework allows for a more logical analysis of indirect and multiple comparisons, which are essential for an NMA; therefore, 60–70% of NMA studies have adopted a Bayesian approach [16, 17]. The differences between the two methodological frameworks are further outlined below. While these two methodological frameworks have different fundamental concepts for approaching the NMA model, they produce almost identical results if the sample size is large [17, 18]. Table 1 explains the common terms used in an NMA with plain words as much as possible, to help readers navigate through the following paragraphs [1–5, 8, 11, 13, 17–28].

**Table 1:** Network meta-analysis concepts and definitions

	Framework	Concept/definition
Indirect treatment comparison (ITC)	Bayesian and frequentist	A comparison of the relative effectiveness across different clinical interventions using data from separate non-head-to-head RCTs
Fixed effects model (FE)	Bayesian and frequentist	The fixed-effect model assumes that there is a true effect size that underlies all the RCTs for each comparison in the network, and that all differences in the observed effect sizes are due to sampling error
Random effects model (FE)	Bayesian and frequentist	The random-effects model assumes that the true effect size can differ from trial to trial
Likelihood function	Frequentist	The likelihood function characterizes the joint probability of the observed data as a function of the parameters of the statistical model
*P* value	Frequentist	The *P* value is the probability of finding a result that is more extreme than the observed result if the null hypothesis was true. *P* values are used to help determine whether to reject the null hypothesis. The smaller the *P* value, the more likely will the null hypothesis be rejected. If the *P* value is smaller than a pre-specified significance level (usually 5%), then the null hypothesis is rejected at this significance level
Confidence interval	Frequentist	A confidence interval provides an estimated range of values that is likely to include an unknown population parameter; it is calculated from the observed data. The confidence level of a confidence interval is the probability that the interval produced by the method used to calculate the confidence interval includes the true value of the parameter; it is usually 95%
Prior distribution	Bayesian	A prior distribution, or prior, of an unknown parameter, usually the mean effect size, is the probability distribution that represents one’s beliefs about this parameter before considering any evidence or observed data
Posterior distribution	Bayesian	The posterior distribution encapsulates all information about an unknown parameter, usually effect sizes, after evidence and observed data are considered. It combines information from the prior distribution and the likelihood function
Posterior summaries	Bayesian	Summary statistics of a posterior distribution; often the mean, median, maximum, minimum, and standard deviation are reported
Credible intervals	Bayesian	A credible interval is an interval within which an unknown parameter value, usually an effect size, falls with a specific probability. It is an interval within a posterior distribution
Ranking probabilities; probability of best treatment; surface under the cumulative ranking area (SUCRA)	Bayesian and Frequentist	Ranking probability is the probability that an intervention is at a specific rank (first, second, etc.) when compared with the other interventions based on a statistic (e.g., mean odds, mean risk, median survival probability). The probability of best treatment is the probability that an intervention is ranked first. The surface under the cumulative ranking curve (SUCRA) is a single number that summarizes the overall ranking of each intervention. Ranking probabilities and SUCRA range from 0 to 100%
Predictive distributions	Bayesian	The predictive distribution is the distribution of possible unobserved (new/ forecasted) values given the observed values
Akaike information criterion (AIC) and Bayesian information criterion (BIC)	Frequentist	The AIC and the BIC are model fit assessments that attempt to explicitly balance model complexity with fit to the observed data. The BIC tends to penalize complex models more compared to the AIC
Deviance information criterion (DIC)	Bayesian	The DIC compares the relative fit of a set of Bayesian models. Like the AIC and the BIC, it is a model selection method which tries to explicitly balance model complexity with fit to the data
Network geometry	Bayesian and Frequentist	The geometry of the network, usually presented as a network plot, consists of a number of nodes (i.e., interventions), a number of edges (i.e., direct comparison evidence), and number of included studies (thickness of the edges)
Transitivity, similarity or exchangeability	Bayesian and Frequentist	The selection of RCTs to formulate the NMA should be based on rigorous criteria and therefore the included RCTs should be similar such that there are no systematic differences between them other than the interventions. That is, the trials in comparison do not differ with respect to the distribution of effect modifiers
Heterogeneity	Bayesian and Frequentist	The variation in trial outcomes between RCTs within the same comparison
Consistency	Bayesian and Frequentist	The degree of agreement between estimates of effect sizes from direct and indirect evidence
Convergence	Bayesian	Samples from the fitted posterior distributions tend to the theoretical posterior distributions as the number of samples becomes adequately large
Effect modifiers	Bayesian and Frequentist	Characteristics that impact the relative clinical intervention effects
Meta-regression	Bayesian and Frequentist	A regression model that models trial-level or arm-level effect sizes with trial-level covariates. It is often used to reduce heterogeneity and inconsistency between RCTs in the network
*I*^2^	Frequentist	The *I*^2^ statistic is the percentage of variation across RCTs that is due to unexplained heterogeneity rather than randomness
*T*^2^	Frequentist	*T*^2^ is the between-studies variance (the variance of the true effect size parameters across all RCTs) parametrized in the random effects model
*τ*^2^	Bayesian	*τ*^2^ is the precision parameter and also the inverse of the between-trial variance parameter in the random effects model. The lower the between-trial variance, the higher is the precision

The Bayesian method combines the known information obtained in the past (prior information) with the present data (likelihood) to calculate the posterior (“post” data observation) probability where the research hypothesis holds [29]. Therefore, the Bayesian method takes a probabilistic approach that allows us to calculate the probability that the research hypothesis holds true, the probability that the true effect size falls within a range—the *95% credible interval (CrI)*, and the ranking probabilities of interventions [8, 29, 30]. Moreover, these probabilities can change depending on prior information [30]. The frequentist method calculates the P value or *the 95% confidence interval (CI)* for rejecting the research hypothesis based solely on present data [7, 8, 17]. Table 2 also highlights differences and similarities between frequentist and Bayesian approaches for NMA [4, 5, 15, 17, 18, 26, 31].

**Table 2:** Differences and similarities between frequentist and Bayesian approaches for network meta-analysis

	Frequentist framework	Bayesian framework
Prior information	Prior information is informally introduced often in the form of supplementary text and is underemphasized	Incorporated within user-specified prior distributions
Basic interpretation	How likely is it to observe the data given a specific parameter value?	How likely is a specific parameter value given the observed data?
Presentation of results	*P* values, confidence intervals, ranking probabilities	Posterior distributions, credible intervals, ranking probabilities
Caveat	*P* values are often misinterpreted as probability that the alternative hypothesis is true. Confidence intervals are often misinterpreted as the probability that the true effect size lies in a particular interval	Priors may be difficult to choose Readers often uncritically overemphasize the subjective component induced by the prior and therefore undermine the quality of the analysis. More complex to conduct
Additional features	Model fit and quality assessed with Akaike information criteria or other similar criteria	Model fit and quality assessed with deviance information criterion

## Illustration of interpretation of NMA results through a recent publication in the Journal of Anesthesia

The Journal of Anesthesia has recently published several NMAs [32–36]. We illustrate the interpretation of NMA results through published studies in the journal. One NMA examined the comparative effectiveness of interventions for managing postoperative catheter-related bladder discomfort (CRBD) [33]. A Bayesian Table 3 NMA including 29 trials with 2841 participants was performed for this study. A total of 14 interventions including placebo were included in the evidence network. The effect sizes of interest were the odds ratio (OR) of CRBD at 0 and 1 h after surgery. The results of a Bayesian NMA are usually presented as estimates of relative effect sizes accompanied by 95% Crl. Relative effect sizes are often ratios (e.g., OR, risk ratio, hazard ratio), and in such cases if the credible interval contains 1, then the comparators are not considered as different in the effect size. If the credible interval lies entirely above or below 1, then the comparators are considered as different in the effect size, and the direction (positive or negative) depends on the nature of the effect size associated with the outcome of interest [5, 37]. For example, the estimated OR of CRBD at 0 h after surgery for ketamine versus placebo is 0.17 with a 95% CrI of (0.04, 0.82), which means the odds of CRBD at 0 h after surgery of ketamine is significantly lower than that of placebo. The 95% CrI also implies the true odds ratio of CRBD at 0 h after survey of ketamine versus placebo has a *95% probability* of being between 0.04 and 0.82. The estimated OR of CRBD at 0 h after surgery of tramadol versus placebo is 0.26 with a 95% CrI of (0.04, 1.73). Since this 95% CrI contains 1, OR of CRBD at 0 h after surgery of tramadol versus placebo has a 95% probability of not being different. A 95% CI under the frequentist approach does not have the same intuitive and practical interpretation, but can only conclude whether the two interventions are statistically different in the effect size at 5% level of significance [37, 38]. A significance level of 5% indicates that there is a 5% risk of concluding that there is a difference when there is actually no difference. That is, if a result is statistically significant, it means it is unlikely to have occurred solely by chance or random factors.

**Table 3:** Available software and statistical packages for network meta-analysis as of December 13, 2021

Statistical package	Framework	Pros	Cons	URL
R	Bayesian and frequentist	Great flexibility, high-quality customizable graphs, free access	Limited user friendliness, steep learning curve, requiring extensive programming knowledge	https://www.rproject.org
WinBUGS/OPENBUGS/JAGS	Bayesian	Great flexibility, free access, accessible through other software (e.g., R)	Limited user friendliness, steep learning curve, requiring extensive programming knowledge, limited graphical functionality	https://www.mrc-bsu.cam.ac.uk/software/bugs/the-bugs-project-winbugs https://www.mrc-bsu.cam.ac.uk/software/bugs/openbugs https://mcmc-jags.sourceforge.io
SAS	Bayesian and Frequentist	Great flexibility	Limited user friendliness, requiring fundamental programming knowledge, cost	https://www.sas.com
Stata	Bayesian and Frequentist	High-quality graphs, variety of analyses available	Limited user friendliness, cost	https://www.stata.com
ADDIS/GeMTC	Bayesian	User friendliness, embeds well-developed methods and techniques that are ready to use	Limited modeling capabilities, limited graphical options	https://gemtc.drugis.org

We illustrate the interpretation of results of a frequentist NMA through a study that examined the effects of individualized positive end-expiratory pressure (PEEP) combined with recruitment maneuver (RM) on intraoperative oxygenation during abdominal surgery [32]. A frequentist NMA including 15 trials with 3634 participants was performed for this study. A total of eight interventions were included in the evidence network. The main effect size of interest was the mean difference in oxygenation index. The results of a frequentist NMA are usually presented as estimates of absolute or relative effect sizes accompanied by 95% Cl. If the Cl does not contain the equalization threshold (e.g., 0 for difference-type effect sizes, 1 for ratio-type effect sizes), the comparators are statistically different in the effect size, and the direction (positive or negative) depends on the nature of the effect size associated with the outcome of interest. For example, the estimated mean difference in oxygenation index between interventions is 145.0 with 95% Cl (87.0, 202.9), which means the oxygenation index of Individualized PEEP + RM is 145.0 higher than that of High PEEP at a 5% significance level. The difference is statistically significantly as the lower edge of 95% CI (i.e., 87.0) is greater than 0.

It is worthwhile to discuss the interpretation of ranking probabilities such as surface under the cumulative ranking area (SUCRA), since these often tend to be misinterpreted in the literature [27, 28, 39]. Table 1 also provides an explanation of these terms. When interpreting these ranking statistics, one should also consider (1) the quality of evidence used in the NMA; (2) confidence in NMA results (further described in the next session); (3) the magnitude of differences in intervention effects; and (4) random chance that may explain any apparent differences between intervention rankings [3, 26, 27, 40]. That is, clinicians and decision makers should not assume an intervention as being “best” simply because it is ranked first, unless the aforementioned aspects of the NMA are fully considered.

## Confidence in NMA results

NMA inherits all challenges present in a conventional pairwise meta-analysis, but with magnified complexity due to the large number of comparisons within the evidence network [37]. To cope with these challenges, NMA adopts a set of assumptions that should be satisfied. The assumptions are (1) similarity or exchangeability, (2) homogeneity and (3) transitivity or consistency [8, 22, 23]. Definitions and concepts of these assumptions are described in detail in Table 1. Typically, if the trial population, trial design and outcome measures are similar for trials that compose the NMA, and that the trials are comparable on effect modifiers (Table 1), these assumptions are adequately satisfied [22, 23]. If one or more assumptions are not satisfied, the NMA becomes inherently biased and in turn yields biased and inaccurate results [41]. To prevent this, remedial measures and adjustments should be applied if appropriate. Methods for assessing NMA assumptions and remedial measures have been developed and widely adopted over the past few years [22, 23]. In addition to these more statistical assumptions, the characteristics of trials in the evidence network that affect the certainty of evidence should be evaluated [42]. These characteristics include risk of bias and publication bias and are often part of the systematic review. These biases usually increase the level of uncertainty of individual trial evidence and subsequently the synthesized evidence in an NMA [3].

In summary, violation of the similarity, homogeneity and consistency assumptions, as well as the presence of any risk of bias and publication bias, affect the overall confidence in the results of an NMA. Therefore, when reviewing a published NMA, one should examine if these issues were identified and how they were dealt with and base one’s confidence in the NMA on these factors. GRADE (Grading of Recommendations, Assessment, Development and Evaluations) is a transparent framework for developing and presenting summaries of evidence [42, 43]. It is the most widely adopted tool for grading the quality of evidence with over 100 organizations worldwide officially endorsing GRADE [42]. GRADE provides a tool to assess the aforementioned statistical assumptions and evidence characteristics for any NMA [42–44]. We recommend reviewing the GRADE assessment of a published NMA if it is available. Other tools to assess the quality of an NMA include checklists published by the National Institute for Health and Care Excellence (NICE), the Professional Society for Health Economics and Outcomes Research (ISPOR), PRISMA and Medical Decision Making (MDM) [3, 26, 40, 45].

## Using individual patient data in a network meta-analysis

Nowadays, as data become easier to collect and assess, we enter an era of “big data” with big data analysis emerging as a new analysis technique in clinical research [46]. We can utilize big data to improve precision of an NMA. An NMA can turn into a big data analysis through incorporating individual patient data (IPD) into its evidence synthesis process [47, 48]. There are benefits of conducting an NMA using IPD over a usual NMA using aggregated trial-level data. If there is interest in patient-specific covariates, either to explain between-study inconsistency or to explore intervention effects in subgroups of patients, using IPD can have much more statistical power than using aggregated trial-level covariates [48]. Furthermore, several studies have shown that the use of IPD in NMA will considerably improve the precision of estimates of intervention effects and regression coefficients in most scenarios [49, 50]. However, IPD may not provide significant improvement to NMAs that have large and dense intervention networks, since the amount of data and evidence are already large and using IPD on top of these will not much improve the precision in the intervention effect estimates [47]. In most NMAs, since IPD may not be available from all eligible RCTs, techniques for combining IPD and aggregated trial-level data into the NMA have been developed Fconsider[47, 50].

## Conclusions

Network meta-analysis has become increasingly popular for synthesizing multiple sources of clinical evidence. It provides the ability to compare multiple clinical interventions where head-to-head trials are not always available by combining direct and indirect evidence from a network of clinical trials. By doing so, it produces less biased and more precise intervention efficacy estimates. While Bayesian and frequentist methods often yield similar results, the two approaches are fundamentally different in theoretical principles and more importantly require different interpretation of the results. The major limitation of NMA is that NMA results hinge on the inherent statistical assumptions of the NMA and the quality of the evidence used in the NMA. The inherent statistical assumptions are strict and often difficult to satisfy, and the quality of evidence used in the NMA are often difficult to uphold. Multiple requirements need to be met for the results to be sound and useful. Therefore, we recommend a thorough, careful, and conservative approach to interpreting and evaluating the results of an NMA. We also recommend using big data analysis techniques to integrate IPD into the NMA to improve the overall quality and precision of the NMA.
